# The photosynthetic pathways of plant species surveyed in Australia’s national terrestrial monitoring network

**DOI:** 10.1038/s41597-021-00877-z

**Published:** 2021-04-01

**Authors:** Samantha E. M. Munroe, Francesca A. McInerney, Jake Andrae, Nina Welti, Greg R. Guerin, Emrys Leitch, Tony Hall, Steve Szarvas, Rachel Atkins, Stefan Caddy-Retalic, Ben Sparrow

**Affiliations:** 1 School of Biological Sciences, The University of Adelaide, Adelaide, South Australia 5005 Australia; 2 Terrestrial Ecosystem Research Network (TERN), University of Adelaide, Adelaide, South Australia 5005 Australia; 3 School of Physical Sciences and the Sprigg Geobiology Centre, The University of Adelaide, Adelaide, South Australia 5005 Australia; 4 CSIRO Agriculture and Food, Urrbrae, South Australia 5064 Australia; 5 School of Life and Environmental Sciences, University of Sydney, Sydney, NSW 2006 Australia

**Keywords:** Biodiversity, Stable isotope analysis, Photosynthesis

## Abstract

The photosynthetic pathway of plants is a fundamental trait that influences terrestrial environments from the local to global level. The distribution of different photosynthetic pathways in Australia is expected to undergo a substantial shift due to climate change and rising atmospheric CO_2_; however, tracking change is hindered by a lack of data on the pathways of species, as well as their distribution and relative cover within plant communities. Here we present the photosynthetic pathways for 2428 species recorded across 541 plots surveyed by Australia’s Terrestrial Ecosystem Research Network (TERN) between 2011 and 2017. This dataset was created to facilitate research exploring trends in vegetation change across Australia. Species were assigned a photosynthetic pathway using published literature and stable carbon isotope analysis of bulk tissue. The photosynthetic pathway of species can be extracted from the dataset individually, or used in conjunction with vegetation surveys to study the occurrence and abundance of pathways across the continent. This dataset will be updated as TERN’s plot network expands and new information becomes available.

## Abstract (summary)

**Table:** 

Measurement(s)	oxidative photosynthetic carbon pathway
Technology Type(s)	digital curation • elemental analysis isotope ratio mass spectrometry
Factor Type(s)	date • geographic location
Sample Characteristic - Organism	Viridiplantae
Sample Characteristic - Environment	terrestrial natural environment
Sample Characteristic - Location	Australia

Machine-accessible metadata file describing the reported data: 10.6084/m9.figshare.14134994

## Background & Summary

The photosynthetic pathway of plants has a substantial impact on species productivity, abundance, and geographic distribution^1–3^. There are three primary photosynthetic pathways. C_3_ photosynthesis is the most common pathway. Plants that use this pathway include cool season grasses, most shrubs, and nearly all trees^4,5^. C_4_ plants include warm-season grasses, many sedges, and some forbs and shrubs^6^. Finally, Crassulacean acid metabolism (CAM) plants most commonly include epiphytes and succulents^7^. C_3_ plants have no special adaptations to prevent photorespiration, an energetically expensive process that occurs when the enzyme rubisco binds with oxygen to produce 2-phosphoglycolate^8–10^. The rate of photorespiration increases with increasing temperature^11^, restricting the photosynthetic capacity of C_3_ plants in warm environments. In contrast, C_4_ and CAM plants possess a series of biochemical, anatomical, and physiological adaptations that concentrate and isolate CO_2_ with rubisco, helping to eliminate photorespiration^6,12^. Consequently, C_4_ and CAM plants more easily live in hot or arid habitats^3,13^.

Global warming is expected to alter the competitive advantage of plants with different photosynthetic pathways^14–16^, changing species distributions and community composition, and leading to significant bottom-up effects on the structure, diversity and function of terrestrial communities^17–19^. Thus, the ecology and evolution of these different pathways has become a focus of recent botanical research^20–22^. Australia is an ecologically diverse continent that includes a wide variety of habitats and climatic zones^23–25^, making it an ideal environment to examine trends in C_3_, C_4_ and CAM distribution^23,26^. However, the photosynthetic pathway of numerous Australian species has not been assessed, and nationally systematic, compatible, and comparable vegetation surveys have not been historically available. The absence of these fundamental data severely limits national terrestrial research capacity.

**Fig. 1:**
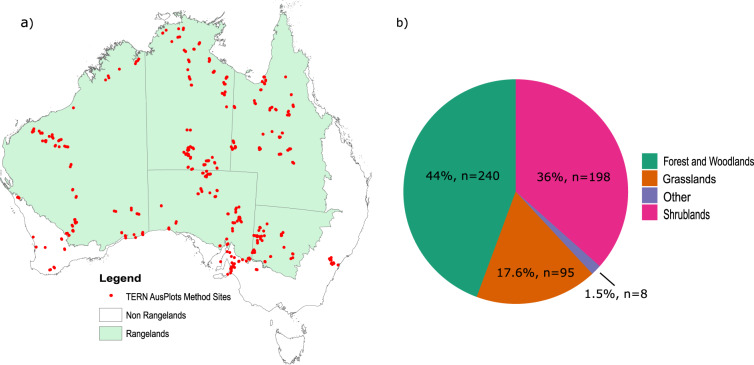
(**a**) Location of TERN Ecosystem Surveillance plots surveyed using the AusPlots Rangelands method from 2011–2017. Areas in green denote rangeland habitat (**b**) number (n) and proportion (%) of TERN Ecosystem Surveillance plots grouped by vegetation type.

Here we provide a dataset that lists the photosynthetic pathways of 2428 species found across Australia. These species were recorded at 541 vegetation survey plots established between 2011 and 2017 (inclusive; Fig. 1). These plots were established by the Terrestrial Ecosystem Research Network (TERN), Australia’s national terrestrial monitoring organisation. TERN is funded by the National Collaborative Research Infrastructure Strategy (NRCIS) and observes, records, and measures critical terrestrial ecosystem parameters and conditions for Australia over time. TERN Ecosystem Surveillance is one of three major branches within TERN, and is responsible for a nation-wide plot survey program. At each plot, TERN records vegetation composition and structural characteristics, and collects a range of soil and plant samples^27,28^. TERN data and resources are made freely accessible to scientists around the globe. The photosynthetic pathway dataset presented here was originally created by TERN to help facilitate research examining the distribution and abundance of C_4_ vegetation in Australia. This dataset will continue to be curated and updated as TERN increases its network of survey plots, and as new research investigates the photosynthetic pathways of terrestrial species.

Photosynthetic pathways were primarily assigned using peer-reviewed literature. We also measured the stable carbon isotope (δ^13^C) values of 540 species that had no recorded pathway. Tissue samples for δ^13^C analysis were acquired from plant specimens collected during TERN plot surveys. Using these techniques, we identified 2048 C_3_, 346 C_4_, 17 C_3_-CAM, and 7 C_3_-C_4,_ 7 CAM, and 4 C_4_-CAM species across all plots. C_4_ species were found in 14 families and 84 genera. Most C_4_ species were Poaceae (228; 65.8%), followed by Cyperaceae (38; 10.9%) and Chenopodiaceae (25; 7.2%). CAM and CAM-facultative species were mainly found in Aizoaceae, Portulacaceae, and Crassulaceae. 14 genera included multiple photosynthetic pathways, specifically *Tetragonia* (Aizoaceae), *Alternanthera* (Amaranthaceae), *Heliotropium* (Boraginaceae), *Polycarpaea* (Caryophyllaceae), *Tecticornia* (Chenopodeceae), *Cleome* (Cleomaceae), *Cyperus* (Cyperaceae), *Euphorbia* (Euphorbiaceae), *Aristida*, *Eragrostis*, *Neurachne*, *Panicum* (Poaceae), and *Tribulus* (Zygophyllaceae). While data can be extracted for individual species, genera, or families, this dataset was designed to be used in conjunction with other TERN products. For example, photosynthetic pathway assignments can be directly combined with matching species records in TERN AusPlots vegetation surveys to obtain data on plant distribution, growth form, height and cover. These records can also be combined with other TERN plot data and products, including climate, soil, and landscape rasters. We expect this dataset will enable work examining patterns in plant occurrence, richness, and abundance, and ecosystem function at local to national scales.

## Methods

The methods used to create this dataset are presented in the following order:

1. The TERN plot-based methodologies used to survey and identify plant species, and preserve plant specimens for stable isotope analysis
2. The procedures used to assign species a photosynthetic pathway using peer-reviewed literature
3. The procedures used to assign species a photosynthetic pathway using stable carbon isotope (δ^13^C) analysis.

### TERN plot survey protocols, species identification, and sample collection

Plant species were identified at 541 one-hectare plots systemically surveyed by TERN between 2011 and 2017 (inclusive). Most TERN plots are located within the Australian rangelands (Fig. 1a). The Australian rangelands encompass 81% of the Australian landmass, and are characterised by vast spaces with highly weathered features, old and generally infertile soils^29^, highly variable rainfall, and diverse and variable plant and animal communities^30^. These areas have traditionally been underrepresented in Australian environmental monitoring programs, which typically focus on more mesic environments and areas closer to large population centres^30^. TERN’s AusPlots Rangelands method^27,28^ and location selection strategy was originally designed to address this underrepresentation by targeting these environments and developing and implementing survey methods that were consistent across the whole of the rangelands. Over time the network has expanded to include sampling in all the major terrestrial environments across the country, including alpine, heathland, and the subtropical systems of the east coast. The dominant vegetation types surveyed at the time of this work were woodlands and savannahs, tussock and hummock grasslands, and shrublands (including chenopod shrublands; Fig. 1b). Climate in TERN plots varies from monsoonal tropics in the north, arid deserts in the centre, to winter-dominant rainfall in the south.

The AusPlots Rangeland method^27,28^ consists of numerous survey modules designed to collect a wide suite of data on soil and vegetation attributes, as well as site contextual information (e.g. erosion, recent fires, etc.). These modules were conceived to provide the data level necessary to study plant community composition and structure, while also ensuring consistency in the collection of samples and data on vegetation, land, and soil characteristics. A complete description of TERN plot survey protocols is detailed in the TERN AusPlots Rangeland manual^27,28^. Only the protocols most relevant to plant surveys, identification, and specimen preservation are documented here.

**Fig. 2:**
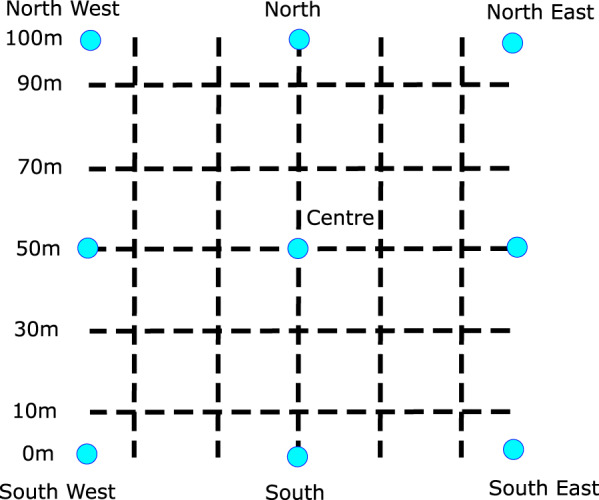
TERN Ecosystem Surveillance plot layout. The corners and centre of the plot (blue dots) are permanently marked with pickets and their locations recorded via GPS. Transects (dashed-lines, 100 m long) are laid in a grid pattern spaced 20 meters apart^28^.

TERN survey plots are 1 ha (100 × 100 m) permanently established sites located in a homogenous area of terrestrial vegetation (Fig. 2). Plots are usually surveyed only once, with an intention to revisit once per decade. Plots are surveyed as seasonal conditions permit, with the aim being to maximise the quality of the plant material collected and facilitate accurate herbarium identifications. Survey teams consist of between 2- and 6 people. A full complement of 6 people would include 1 to 2 people performing the vegetation survey modules, 1 to 2 people performing the soil survey modules, and the remaining team members undertaking other components of the Ausplots Rangelands method, such as recording site contextual information. The duration of each survey is variable and dependent on the density and diversity of the vegetation. Plot selection and orientation avoids major anthropogenic influences (such as roads, cattle yards, fences, bores, etc.). Ten transects (100 m long) are laid out within each plot in a grid pattern. Parallel transects running north to south are spaced 20 meters apart located at 10, 30, 50, 70, and 90 m both north and east from the SW corner (Fig. 2). Each plot is given a unique alphanumeric identifier that indicates the location of the plot, specifically its state (e.g. Western Australia, South Australia, Northern Territory, etc.) and Interim Biogeographic Regionalisation for Australia (IBRA) version 7 bioregion^31^, and a sequential number based on the number of plots in that bioregion. The date of the survey and GPS co-ordinates are also recorded for each plot.

**Fig. 3:**
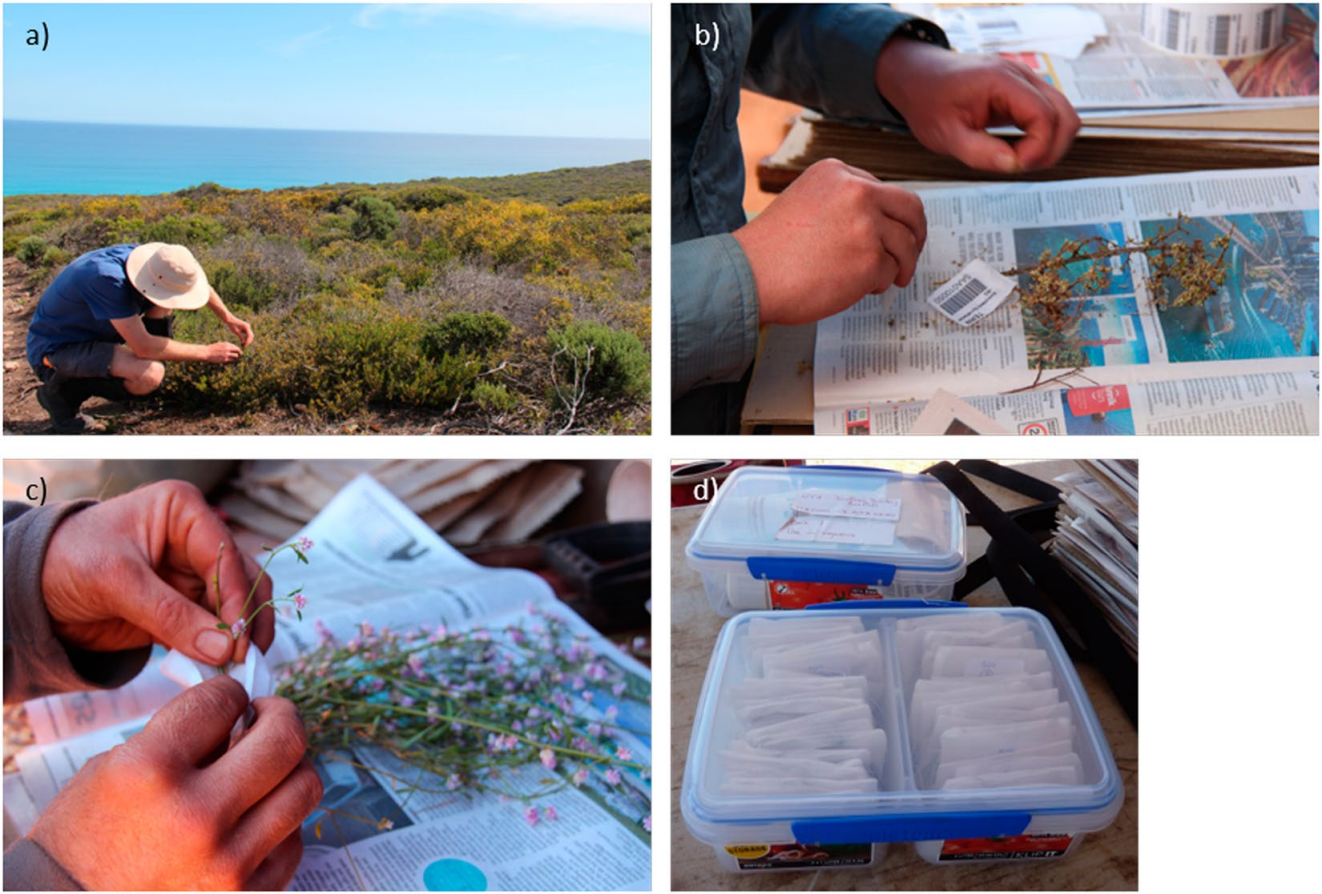
Collection procedures of vascular flora by TERN Ecosystem Surveillance team. (**a**) Collection of vascular flora by ground observers. (**b**) Voucher specimens are collected with enough material to fill an A3 size herbarium sheet, pressed, and ultimately sent to local herbaria for identification, (**c**) subsamples of each voucher specimen are collected from the main voucher sample to enable stable isotope analysis, the subsample is placed in a gauze “teabag” and (**d**) then sealed in a plastic container with 1 cm depth of silica granules (Photo Credit: TERN Ecosystem Surveillance program).

Recording, collection, and identification of vascular flora is undertaken by specially trained members of the field survey team. One ground observer is tasked to perform line intercept transects. This ground observer records the species and substrate at each point (1 m) along each transect, resulting in survey data at 1010 points per plot. These point-intercept data are collected to calculate species cover (%) and other metrics. A second ground observer collects specimens of each vascular plant species in the plot, with enough material to fill an A3 size herbarium sheet (Fig. 3a,b). These members of the survey team work together to ensure the presence of each vascular plant species is recorded and enough specimens are collected. Each specimen ideally contains flowers or buds, leaves, fruit, and bark (for trees) to help enable identification. Each specimen is then tagged with a unique alphanumeric voucher barcode. All field and voucher data are recorded using a purpose-built app on a tablet to streamline data and sample collection^32^. The voucher specimen is ultimately delivered to a local herbarium for identification.

Subsamples of each voucher specimen are collected from the main voucher sample to enable stable isotope and molecular analysis (Fig. 3c). These subsamples are ideally free from disease, insect, or fungal contamination. The subsample is placed in a synthetic gauze ‘teabag’ and given its own unique alphanumeric barcode, referred to as the ‘primary genetic barcode’, which is linked to the date, plot, state, and voucher specimen from which it was collected. All teabags for a plot are then sealed in an air-tight, plastic container with 1 cm depth of silica granules (Fig. 3d). The container is stored in a cool location out of direct light for the duration of the survey. Upon return from the field, teabags are stored in dark conditions at room temperature at TERN facilities at the University of Adelaide (Adelaide, Australia). The silica granules are changed regularly until the samples are dehydrated and then replaced as necessary to keep the samples dry.

### Photosynthetic pathway assignment

All TERN plant data were processed in the R statistical environment^33^ using the *ausplotsR* package^34,35^. The *ausplotsR* package was created by TERN to enable the live extraction, preparation, visualisation, and analysis of TERN Ecosystem Surveillance monitoring data. A list of all vascular plant species at each TERN plot was extracted using the *get_ausplots* function. This produced an initial list of 4002 unique records. Scientific names for each record are provided by herbaria and are the most commonly used names in the state where the voucher specimen was collected. However, scientific names sometimes vary between states due to jurisdictional differences in taxonomy and nomenclature. TERN Ecosystem Surveillance uses the scientific names as determined by the herbaria as the point of truth in all its analysis and data sets. State herbaria identify species to the lowest possible taxonomic level. Specimens that were only identified to the family or genus level were excluded from the photosynthetic pathway dataset. Hybrids were also excluded from the final species list. Varieties and subspecies were assumed to have the same photosynthetic pathway^36^, therefore photosynthetic pathways were assigned to the species (i.e. *Genus species*) rank. This process of elimination generated a final list of 2613 unique species.

To assign each species a photosynthetic pathway, scientific names were first cross-referenced against well-known plant trait databases including Kattge, *et al*.^24^, Osborne, *et al*.^36^, and Watson and Dallwitz^37^. We then conducted literature searches of the remaining unassigned species via Google Scholar with combinations of the key words “C_3_”, “C_4_”, “CAM”, “photosynthesis” and “photosynthetic pathway”. We used a total of 34 peer-reviewed sources to assign species photosynthetic pathways (Table 1). If species-specific information was not available, but the species belonged to a genus known to be exclusively C_3_, C_4_ or CAM it was assigned to that pathway (e.g. *Acacia* spp., *Eucalyptus* spp. are presumptive C_3_). Using these combined strategies, 1888 species were assigned a photosynthetic pathway. Discrepancies between sources were rare (total of 5). In cases where species were assigned different photosynthetic pathways by different sources, the photosynthetic pathway from the source that provided the best direct evidence to support the assignment was selected. If it was not possible to assign a photosynthetic pathway using published sources or presumptive reasoning, then that species was selected for stable carbon isotope analysis.

**Table 1:** List of databases and peer-reviewed literature used to assign species in TERN plots a photosynthetic pathway.

Source	Number of species assigned a photosynthetic pathway
Besnard, *et al*.^60^	1
Bohley, *et al*.^61^	1
Bruhl and Wilson^62^	8
Caddy-Retalic^63^	154
Carolin, *et al*.^64^	2
Clayton, *et al*.^65^	3
D’andrea, *et al*.^66^	1
Ehleringer and Monson^67^	3
Feodorova, *et al*.^68^	1
Guillaume, *et al*.^69^	9
Hancock, *et al*.^45^	7
Herppich and Herppich^70^	1
Holtum, *et al*.^71^	2
Holtum, *et al*.^72^	1
Horn, *et al*.^73^	10
Kadereit, *et al*.^74^	1
Kattge, *et al*.^24^	1013
Koch and Kennedy^75^	1
Madhusudana Rao, *et al*.^76^	1
Metcalfe^77^	3
Osborne, *et al*.^36^	6
Pate, *et al*.^78^	8
Sage^5^	657
Sage, *et al*.^25^	27
Sayed^7^	2
Schmidt and Stewart^79^	1
Taylor, *et al*.^80^.	1
Thiede and Eggli^81^	2
Ting^82^	1
Watson and Dallwitz^37^	637
Watson and Dallwitz^83^	2
Winter^12^	1
Winter, *et al*.^84^	2
Winter, *et al*.^54^	3

Sometimes multiple sources were used to justify the photosynthetic pathway assignment of a single species, as a result the total ‘Number of species assigned a photosynthetic pathway’ is greater than the number of unique species recorded in TERN plots.

### The stable carbon isotope values of C_3_, C_4_, and CAM plants

The stable carbon isotope values of C_3_ plants range from −37‰ to −20‰ δ^13^C (mean = ~−27‰), while the values of C_4_ plants range from −12‰ to −16‰ δ^13^C (mean = ~−13‰)^38,39^. Therefore, for species where either a C_3_ or C_4_ pathway was possible (e.g. Poaceae), plants with δ^13^C values <−19‰ were designated C_3,_ and plants with δ^13^C values >−19‰ were designated C_4_^26^. Full CAM plants, or plants in which CAM is strongly expressed, have isotope values of >−20‰, and thus can be distinguished from C_3_ plants using δ^13^C^39,40^. However, CAM photosynthesis almost always co-exists with the C_3_ pathway (C_3_-CAM)^12^. The isotope values of C_3_-CAM plants are correlated with the proportion of carbon that is obtained during light and dark periods. As a result, C_3_-CAM δ^13^C values are highly variable (approximately −13‰ to −27‰) and are dependent upon the species, its developmental stage, and/or the time of day and conditions during which the plant was sampled^40–42^. For example, the CAM pathway is often upregulated during periods of stress, such as drought^43,44^. Therefore, although the δ^13^C of wild plant samples can be used to indicate CAM potential, stable isotope values are not a reliable way to distinguish CAM and C_4_, identify CAM when it is weakly expressed, or a definitive method to discriminate C_3_ and C_3_-CAM plants^41,42^. To confirm the presence of CAM, additional measures of other physiological and biochemical variables are usually required^45^. With this limitation in mind, for genera with previously confirmed C_3_-CAM potential, we followed past authors and tentatively denoted plants with a δ^13^C value >−20‰ as CAM, −21‰ to −24‰ as potentially C_3_ + CAM, and plants <−24‰ as C_3_^40,45,46^.

#### Isotope analysis

540 species were selected for stable isotope analysis. The remaining 184 unassigned species were not included in δ^13^C analysis because no suitable tissue samples were available. TERN plant tissue samples were identified and selected using the *ausplotsR* package. Each species record is associated with a full list of the available silica-dried tissue samples. One sample was selected for stable isotope analysis based on overall condition and availability (i.e. the amount of sample available from a given plot).

A 2 g subsample of material was taken from each silica-dried tissue sample. Each subsample was placed in an Eppendorf tube with two small ball bearings and pulverised for approximately one minute at 30 htz using a Retsch Mixer Mill. If samples had not homogenised during this initial process, samples were transferred to a stainless-steel ball-mill grinder and were ground for a further one minute at 30 htz. Sample preparation procedures were performed at the Mawson Analytical Spectrometry Services (MASS) Facility, University of Adelaide. An initial group of 378 samples were analysed for stable isotopes at both MASS and the Stable Isotope Facility at the Waite Campus of CSIRO in 2019. A subsequent group of 162 plant samples were analysed in 2020 at MASS.

### Stable carbon isotope analysis at CSIRO

2 to 2.5 mg of powdered plant samples were weighed into tin cups and analysed for δ^13^C using a continuous flow isotope ratio mass spectrometer (IRMS Delta V, ThermoBremen, Germany) equipped with an elemental analyser (Flash EA, Thermo, Bremen, Germany). Stable isotope ratios were expressed in δ notation as deviations from a standard in parts per mil (‰):

1 $${\delta }^{13}C=[({R}_{{\rm{s}}{\rm{a}}}/{R}_{{\rm{r}}{\rm{e}}{\rm{f}}})-1]\times 1000$$

where *R*_sa_ is the ratio of abundances of ^13^C/^12^C in the sample, and *R*_ref_ is this ratio in the reference gas^47^. δ^13^C was reported relative to the standard Vienna Pee Dee Belemnite (VPDB). See the “Technical Validation” section for normalisation methods and precision estimates.

### Stable carbon isotope analysis at MASS, University of Adelaide

Like the procedures at CSIRO, 2 to 2.5 mg of powdered plant samples were weighed into tin cups and analysed for δ^13^C using a continuous flow isotope ratio mass spectrometer (Nu Horizon, Wrexham, UK) equipped with an elemental analyser (EA3000, EuroVector, Pavia, Italy). Stable isotope ratios were expressed in δ notation as deviations from a standard in parts per mil (‰) using Eq. 1. δ^13^C was reported relative to the standard Vienna Pee Dee Belemnite (VPDB). See the “Technical Validation” section for normalisation methods and precision estimates. Once all stable isotope analysis was complete, a final dataset was compiled that listed the photosynthetic pathway of 2429 plant species detected in TERN plots^47^.

## Data Records

All data records are stored in the TERN Geospatial Catalogue repository and can be found via the TERN Data Discovery Portal^47^. Data has been released under a CC‐BY Creative Commons license (https://creativecommons.org/licenses/by/4.0/), which allows reuse with attribution. Any work or publications using these data should cite this descriptor and, if applicable, the original sources (Table 1). The data set is comprised of two data tables and one data descriptor file that defines the values in the two data tables (Table 2). All tables and files are in MS Excel (.xlsx). The first table contains a list of each species and its photosynthetic pathway. It specifies the method used to determine the photosynthetic pathway (i.e. peer-reviewed literature, inferred from lineage, or δ^13^C analysis), as well as the peer-reviewed source or δ^13^C value of the tested specimen, as applicable. The plot number, location, and date that specimens were collected, the facility where the stable isotope analysis was conducted, and any replicate δ^13^C values are also provided. Details on commonly used species name synonyms are also listed (see Usage Notes for details). Any discrepancies in photosynthetic pathway assignments between sources, or notes about the need for further testing to confirm tentative assignments, are also recorded for each species. The second table includes a list of all the peer-reviewed sources used to create this dataset. Updates to the dataset will be managed through the TERN Geospatial Catalogue by creating a new version of the dataset. As TERN continues to expand its plot network, we will aim to include new species on an annual basis. We will also re-evaluate species taxonomy and photosynthetic pathways as new information becomes available.

**Table 2:** Description of database “The photosynthetic pathways of plant species surveyed in TERN Ecosystem Surveillance plots” with file locations.

Source	Document Name	n. records	Data Description	Methods
Link	Plant Photosynthetic Pathway	2428	Photosynthetic Pathway of vascular plant species detected in TERN Ecosystem Surveillance plots	Literature search and stable isotope analysis
Link	List of Studies	34	Alphabetical list of references for species photosynthetic pathways	Literature Search
Link	Data Descriptor	26	Alphabetical list of descriptions for each data column in the “Plant Photosynthesis Pathway” data table	NA

## Technical Validation

TERN Ecosystem Surveillance plot surveys have been performed by different individuals and teams, which has the potential to introduce errors in plant identification in the field by ground observers. For this reason, all collections are given a temporary field name identification and assigned a permanent primary genetic barcode that is associated with a physical plant sample. Each data point and sample are tracked and recorded using the primary genetic barcode, which ensures each data point in the transect is correctly associated with a physical sample for later identification. TERN data is not published until the temporary field names are confirmed or corrected by expert local taxonomists at regional herbaria. Prior to publication of plot plant data, each species is cross-referenced against the Australian Plant Census (https://www.anbg.gov.au/chah/apc/) to confirm correct nomenclature. The whole database is also routinely compared to the Plant Census to detect changes in taxonomy over time.

Photosynthetic pathway assignments obtained from published sources have already been subject to scientific scrutiny and are well-validated. The assumption that all species within a given genus possess the same photosynthetic pathway is realistic in most circumstances^3^. However, our own work and the work of others has identified multiple exceptions. C_4_ and CAM photosynthesis have independently evolved multiple times across dozens of lineages^48,49^, which introduces the potential for misclassifications. To minimise this potential source of error, all species within a given family that are known to include C_4_ species were targeted for δ^13^C analysis. We targeted species in the families Aizoaceae, Asteraceae, Boraginaceae, Caryophyllaceae, Chenopodiaceae, Euphorbiaceae, Poaceae, Portulacaceae, and Zygophyllaceae. We recognize that Chenopodiaceae is now a subfamily of Amaranthaceae; however, chenopods have traditionally been examined as a separate family in past C_4_ analysis^50–52^. Therefore, to enable consistent comparisons with previous work and datasets we distinguished Chenopodiaceae independent of Amaranthaceae. As previously discussed, CAM or C_3_-CAM photosynthesis is particularly difficult to identify using δ^13^C, therefore any CAM or C_3_-CAM designations based on δ^13^C values should be considered tentative and warrant further investigation. Special mention should also be made of the genus *Portucula* (Portulacaceae). Traditionally considered a C_4_ genus, recent evidence has found some *Portucula* species have CAM potential^53,54^. Until species-specific information becomes available, most *Portucula* species in the dataset have been assigned to the C_4_ pathway, but the possibility of C_4_-CAM should be considered.

Stable isotope analysis was performed at two different laboratories over multiple years, therefore technical validation needs to be considered. Each laboratory measured plant δ^13^C using well-established analytical techniques. All samples where corrected for instrument drift and normalized according to reference values^55^ using a combination of certified and in-house calibrated standards (Table 3). For the stable isotope analysis conducted at CSIRO in 2019, all samples were normalized using a multipoint linear regression, where the slope and intercept are used to correct the isotope data on the δ^13^C_VPDB_ scale^56^. Using the multipoint normalization procedure, measured δ values for the analysed standards are plotted on the x-axis, and the “true” accepted δ values expressed on the δ^13^C_VPDB_ scale are plotting on the y-axis. These points create a regression line (Eq. 2) that covers the range of δ values:

2 $${\delta }_{Spl}^{T}=a\times {\delta }_{Spl}^{M}+b$$

Where *a* is the slope and *b* is the intercept. To normalize data, the measured δ value of the sample (*δ*^*M*^_*Spl*_) is multiplied by the slope and the value of the intercept is added. Stable carbon isotope values had uncertainties of ≤0.77*‰ δ*^13^C based on repeat analysis of all the standards (n = 141). The mean and standard deviation of the absolute difference between replicate samples (10% of all samples) was 0.20 ± 0.34‰ δ^13^C.

**Table 3:** List of standards (and their verified values) used to correct for instrument drift and normalize the δ^13^C of plant samples analyzed at the Stable Isotope Facility at the Waite Campus of CSIRO and the Mawson Analytical Spectrometry Services (MASS) Facility, University of Adelaide.

Standard	Verified δ^13^C Value (‰)	Facility
USGS-40	−26.39	CSIRO/MASS
High Organic Sediment Standard OAS	−28.85	CSIRO
Wheat Flour Standard OAS	−26.43	CSIRO
Sorghum Flour Standard OAS	−13.78	CSIRO
Glycine	−31.20	MASS
Glutamic Acid	−16.72	MASS
Triphenylamine	−29.20	MASS
USGS 41	−37.63	MASS

USGS-40 is a certified standard, all others were calibrated in-house by each facility.

MASS standards were calibrated using a two-point correction^57^:

3 $${{\rm{\delta }}}_{{\rm{sa,c}}}={{\rm{\delta }}}_{{\rm{std1}}}+\left[\left({{\rm{\delta }}}_{{\rm{sa,i}}-{{\rm{\delta }}}_{{\rm{std1,m}}}}\right)\right]/\left({{\rm{\delta }}}_{{\rm{std2,m}}-{{\rm{\delta }}}_{{\rm{std1,m}}}}\right)$$

where δ_sa,c_ is the corrected value of the measurement, δ_std1,m_ and δ_std2,m_ are the measured values of the standards, and δ_std1_ and δ_std_ are the known values of the standards. For the isotope analysis conducted at MASS in 2019, isotope values had uncertainties of ≤0.31*‰ δ*^13^C based on repeat analysis of all the standards (n = 30). For the isotope analysis conducted at MASS in 2020, isotope values had uncertainties of ≤0.09*‰ δ*^13^C based on repeat analysis of all the standards (n = 75). The mean and standard deviation of the absolute difference between replicate samples (10% of all samples) in 2020 was 0.24 ± 0.48‰ δ^13^C. Given the broad but unique range of isotope values exhibited by C_3_ and C_4_ species, small deviations in values between laboratories are not likely to affect photosynthetic pathway assignment.

## Usage Notes

All photosynthetic pathways assignments in this dataset are available in the public plant trait database ‘Austraits’, which aggregates trait values for Australian plants. Site descriptions and complete species and specimen lists can be freely accessed for all TERN plots via the TERN *ausplotsR* package (available via CRAN and with the latest development version and patches at https://github.com/ternaustralia/ausplotsR)^34,35^, or the TERN Data Discovery Portal (https://portal.tern.org.au/). As previously described, *ausplotsR* allows users to directly access all TERN plot-based data on vegetation and soils across Australia^34,35^. It also provides functions that calculate and visualise species presence, richness and cover (%) at all TERN plots. The photosynthetic pathway dataset presented here was designed to be easily combined with TERN *ausplotsR* species distribution data to investigate national distribution patterns of different photosynthetic pathways. As an example, we have provided sample code for the R statistical environment to demonstrate how the TERN photosynthetic pathway dataset presented here and % species cover calculated at TERN plots can be combined to calculate C_4_ plant cover (relative to C_3_) across Australia, and relate relative C_4_ cover values to changes in climate and local factors. As detailed in Supplementary File 1, simple functions in *ausplotsR* can quickly calculate % species cover at each TERN plot, and then each species in each plot can be assigned its correct photosynthetic pathway using the TERN photosynthetic pathway dataset. This enables the calculation of relative C_4_ plant cover at each plot. Relative C_4_ cover can then be regressed against climate and local parameters by using TERN plot coordinates to extract site-specific environmental data from other national climate^58^ and soil^59^ rasters.

Additional TERN data infrastructure can be found via the TERN Data Discovery Portal. For more information and tutorials on how to access TERN data, visit www.tern.org.

As previously discussed, scientific names for species in the TERN database are provided by state herbaria and are the most commonly used names in a given state. However, valid scientific names may vary between states due to differences in nomenclature (although this is rare). TERN Ecosystem Surveillance uses the scientific names as provided by the local herbaria as the point of truth in all its analysis and datasets. To enable the integration of this dataset with other data records, where there are known nomenclature issues between jurisdictions, we have notated alternative synonyms in the species name comments field of Table 1 in the dataset. When using this dataset, users should take care to select the most relevant synonym for their work.

## Supplementary information

Supplementary File 1

## Data Availability

No custom code was used in this analysis. Examples of how to combine this photosynthetic pathway dataset with other TERN data infrastructure in the R statistical environment has been provided in the supplementary material (Supplementary File 1).
